# Trichostatin A-Mediated Epigenetic Transformation of Adult Bone Marrow-Derived Mesenchymal Stem Cells Biases the *In Vitro* Developmental Capability, Quality, and Pluripotency Extent of Porcine Cloned Embryos

**DOI:** 10.1155/2015/814686

**Published:** 2015-03-18

**Authors:** Marcin Samiec, Jolanta Opiela, Daniel Lipiński, Joanna Romanek

**Affiliations:** ^1^Department of Biotechnology of Animal Reproduction, National Research Institute of Animal Production, Krakowska 1 Street, 32-083 Balice n. Kraków, Poland; ^2^Department of Biochemistry and Biotechnology, Poznań University of Life Sciences, Dojazd 11 Street, 60-632 Poznań, Poland; ^3^Institute of Human Genetics, Polish Academy of Sciences, Strzeszyńska 32 Street, 60-479 Poznań, Poland

## Abstract

The current research was conducted to explore the *in vitro* developmental outcome and cytological/molecular quality of porcine nuclear-transferred (NT) embryos reconstituted with adult bone marrow-derived mesenchymal stem cells (ABM-MSCs) that were epigenetically transformed by treatment with nonspecific inhibitor of histone deacetylases, known as trichostatin A (TSA). The cytological quality of cloned blastocysts was assessed by estimation of the total cells number (TCN) and apoptotic index. Their molecular quality was evaluated by real-time PCR-mediated quantification of gene transcripts for pluripotency- and multipotent stemness-related markers (*Oct4, Nanog*, and *Nestin*). The morula and blastocyst formation rates of NT embryos derived from ABM-MSCs undergoing TSA treatment were significantly higher than in the TSA-unexposed group. Moreover, the NT blastocysts generated using TSA-treated ABM-MSCs exhibited significantly higher TCN and increased pluripotency extent measured with relative abundance of *Oct4* and *Nanog* mRNAs as compared to the TSA-untreated group. Altogether, the improvements in morula/blastocyst yields and quality of cloned pig embryos seem to arise from enhanced abilities for promotion of correct epigenetic reprogramming of TSA-exposed ABM-MSC nuclei in a cytoplasm of reconstructed oocytes. To our knowledge, we are the first to report the successful production of mammalian high-quality NT blastocysts using TSA-dependent epigenomic modulation of ABM-MSCs.

## 1. Introduction

The efficiency of somatic cell cloning in pigs, which is measured with the rate of embryos developing up to blastocyst stage or the rate of born offspring in relation to the number of reconstructed oocytes, remains disappointingly low. Moreover, despite tremendous improvement of somatic cell nuclear transfer (SCNT) technique in pigs, high early-, mid-, and late-gestation mortality rates of nuclear-transferred embryos/fetuses as well as numerous malformations of resultant cloned offspring still often appear in this species. Incomplete and aberrant reprogramming of epigenetic memory of somatic cell nuclei in preimplanted nuclear-transferred (NT) embryos is one of the most important factors that limit cloning effectiveness in this species [1–4].

The process of epigenomically dependent reprogramming is related to the stable erasure (vanishing) of donor cell nuclear DNA-epigenetic status and turning back (molecular nulling) the somatogenic “transcriptional and translational clock.” This contributes to recapitulation of a particular program of the embryonic genome expression, which is induced by the reestablishment of the embryo cell genome-associated methylation and embryo cell chromatin-associated acetylation patterns [5–7]. SCNT-linked problems are hypothesized to result from aberrant gene dedifferentiation of somatic cell nuclei at the levels of epigenomic, genomic, and molecular memory. The mechanism underlying the dedifferentiation process of donor nuclei is the cessation of their own gene expression and reversal of the differentiated (specialized) somatic nucleus to a totipotent/pluripotent embryonic (undifferentiated/unspecialized) state within the host ooplasm and cytoplasm of cleavage descendant blastomeres of NT embryos [8–10]. In turn, impaired restoration (reestablishment) of the totipotency/pluripotency of embryonic cell lines in the first phase of epigenetic reprogramming (i.e., gene dedifferentiation) during preimplantation development by the blastocyst stage may trigger disadvantageous alterations in the second phase of donor nuclear reprogramming. These are connected with improper redifferentiation of somatic cell-inherited genes throughout postimplantation fetal/placental development. Additional work is needed to determine whether failures in the early-stage reprogramming are magnified downstream in development [11–13].

*In vitro* cultured fibroblast cells, which had been derived from the dermointegumentary tissue of fetuses and adult specimens, are the commonly used source of nuclear donor cells in the pig cloning procedure [14–18]. The degree of molecular and epigenetic differentiation of these cells that is related both to the advanced methylation profile of DNA cytosine residues and to the lysine deacetylation profile of histones forming nucleosomal core of nuclear chromatin often seems to make the converting of the abovementioned covalent modifications back to a totipotent state of embryonic (zygotic) cells impossible. This leads mainly to decrease in the abilities of differentiated fibroblast cells for supporting the* in vitro* development of cloned embryos to the blastocyst stage [1, 19]. As a rule, the percentage of blastocysts originating from the porcine oocytes reconstructed with fetal or adult cutaneous fibroblast cell nuclei oscillates from 10% to 30% [20–23].

It is hypothesized that the use of undifferentiated mesenchymal stem cells (MSCs) isolated from adult bone marrow, which are characterized by the high multipotency level and genomic/epigenomic plasticity, allows increasing the preimplantation developmental potential of mammalian cloned embryos. As compared to hematopoietic stem cells, the MSCs exhibit the lower activity of histone deacetylases (HDACs) and DNA methyltransferases (DNMTs), which results in hyperacetylation of histone lysine moieties and demethylation of DNA cytosine residues [24–27]. The last two processes affect the inhibition of transcriptional suppression of many regions in the nuclear genome of multipotent MSCs. All these properties are responsible for the high susceptibility of the MSC nuclei to correct and complete epigenetic reprogramming in the cytoplasm of NT embryo blastomeres. Therefore, the genome of undifferentiated stem cells may be more easily reprogrammed to resemble the genome of the zygote, which may make stem cells more efficient as nuclear donors in the somatic cell cloning [28–30]. Generally, porcine NT embryos reconstituted with adult bone marrow-derived mesenchymal stem cells displayed considerably higher blastocyst formation rates than those reconstituted with adult cutaneous or fetal fibroblast cells [31–33].

Transcriptional activity of somatogenic nuclear genome during embryo pre- and/or postimplantation development as well as fetogenesis is correlated with the frequencies for spatial remodeling of chromatin architecture and reprogramming of cellular epigenetic memory. This former and this latter process include such covalent modifications as demethylation/*de novo* methylation of DNA cytosine residues and acetylation/deacetylation as well as demethylation/remethylation of lysine residues of nucleosomal core-derived H3 and H4 histones [2, 30, 34, 35]. The level of progression for the processes of epigenetic genome-wide alterations that are mediated by DNA methyltransferases (DNMTs 1o and 3a/3b) and histone deacetylases (HDACs) can be modulated (i.e., reversed)* via* exogenous inhibitors of these enzymes throughout* in vitro* culture of nuclear donor cells and/or cloned embryos [19, 36–39]. Moreover, the use of the artificial modifiers of epigenomically conditioned gene expression leads to the inhibition of both chromatin condensation and transcriptional silencing of the genomic DNA of cultured somatic cells that are applied as a source of donor nuclei for the reconstruction of enucleated oocytes and subsequent generation of cloned embryos. The members of these epigenetic modifiers are 5-aza-2′-deoxycytidine (5-aza-dC) [37, 39–41],* S*-adenosylhomocysteine (SAH) [42], trichostatin A (TSA) [6, 19, 36, 38, 43], valproic acid/sodium valproate (VPA/SV) [44, 45], 6-(1,3-dioxo-1*H*,3*H*-benzo[de]isoquinolin-2-yl)-hexanoic acid hydroxyamide called scriptaid [46–49], sodium butyrate (NaBu) [50, 51],* m*-carboxycinnamic acid* bis*hydroxamide (CBHA) [30, 52], and oxamflatin [29, 53]. The onset of chromatin decondensation and gene transcriptional activity is evoked by nonspecific/nonselective blocking of the activity of either DNMTs by 5-aza-dC and SAH [41, 42] or HDACs by the TSA, VPA, scriptaid, NaBu, CBHA, and oxamflatin [30, 36, 44, 48, 51, 53]. Such exogenous epigenomic modulation (epigenetic transformation) of nuclear donor cells or cloned embryos may facilitate and accelerate the reprogrammability for gene expression of donor cell nuclei that have been transplanted into cytoplasmic microenvironment of recipient oocytes. Subsequently, these cell nuclei undergo the proper dedifferentiating and faithful reestablishing of the epigenetically dependent status of their transcriptional activity during the preimplantation development of cloned embryos [43, 54, 55].

To increase the developmental capacity of porcine cloned embryos by enhancement of donor cell nuclear reprogrammability, a nonselective inhibitor of HDACs, designated as TSA, was used for epigenetic transformation of adult bone marrow-derived mesenchymal stem cells (ABM-MSCs) that provided a source of nuclear donor cells. In the current investigation, we focused on determining the impact of TSA-dependent epigenomic modulation of cultured MSCs on the somatic cell cloning efficiency measured with* in vitro* developmental capability of NT pig embryos and their cytological quality at the blastocyst stage that was evaluated on the basis of both total blastomere number and apoptotic index estimated by TUNEL analysis. Furthermore, we examined whether exposure of MSCs to TSA affects the pluripotency status of cloned pig blastocysts. For this reason, the quantitative expression profiles for gene transcripts encoding such proteins as pluripotency-related markers (*Oct4* and* Nanog*) and multipotent stemness-associated marker (*Nestin*) were evaluated in nuclear-transferred embryos reconstructed with epigenetically modified MSCs. To the best of our knowledge, this report is the first in which the effect of TSA-mediated epigenetic transformation of nuclear donor ABM-MSCs on the extracorporeal developmental competences of porcine cloned embryos, their cytological quality, and quantitative pluripotency profile at the DNA transcription level was comprehensively explored.

## 2. Materials and Methods

### 2.1. Animals

Four outbred Polish Large White (PLW) pigs of either sex, weighing approximately 20 kg each, were maintained under conventional conditions in the pigsty of the Department of Biotechnology of Animal Reproduction from National Research Institute of Animal Production in Balice, Poland. The veterinary care was provided. All animal procedures were approved by the Local Animal Care Ethics Committee Number II in Kraków.

### 2.2. Chemicals and Supplies

The reagents used in the present experiments were purchased from Sigma-Aldrich (Poznań, Poland), unless otherwise indicated.

### 2.3. Recovery and* In Vitro* Culture of Porcine Mesenchymal Stem Cells

Mesenchymal stem cells (MSCs) were isolated from the pig bone marrow as described by Opiela et al. [56, 57]. Briefly, the bone marrow was aspirated under general anesthesia. Bone marrow samples were placed in phosphate-buffered saline (PBS; Biomed, Lublin, Poland) (1 : 1), and 6 mL of this solution was layered over 3 mL of Ficoll-Paque (Stem-cells Technologies, USA). Following centrifugation, the mononuclear cell fraction was collected and rinsed twice with PBS. The pelleted cells were transferred to 75 cm^2^ tissue culture flasks (T75) filled with 17 mL of MSC growth/expansion medium (Dulbecco's Modified Eagle's Medium; DMEM) that was formulated to contain low concentration (1 mg/mL) of* D*-glucose and enriched with 10% fetal bovine serum (FBS) and 1% antibiotics/1% Glutamax (Invitrogen, USA). The culture medium was replenished on the next day, and the adherent cells were allowed to form colonies until reaching confluence. The cell colonies were harvested by trypsin/EDTA treatment and passaged. Afterwards, the cells were seeded* de novo* at a concentration of 0.25 to 0.5 × 10^6^ into one T75 flask. To confirm the mesenchymal stemness origin of isolated cells, flow cytometry-based detection of positive and negative expression of specific surface cluster of differentiation (CD) antigens and* ex vivo* differentiation of established MSC lines into adipocytes and osteocytes were accomplished as described by Opiela et al. [56, 58].

### 2.4. Simultaneous Cell Cycle Synchronization and Epigenomic Modulation (Epigenetic Transformation) of Porcine MSCs prior to SCNT

Before trypsin-mediated detachment and use for somatic cell cloning, the cryopreserved permanent MSC lines (between passages 1 and 2) that had been established from the primary cultures originating from bone marrow biopsies of the prepubertal boars or gilts were thawed and cultured* in vitro* in DMEM medium enriched with relevant supplementations. The mitotic cycle of MSCs was synchronized at G1/G0 stages through 24 to 48 h contact inhibition of their proliferative activity after reaching the total confluence state under culture conditions in the medium enriched with 10% FBS. During artificial synchronization of cell cycle, MSCs were epigenetically transformed by 24 h exposure to 50 nM TSA.

### 2.5. Preparation of Porcine Nuclear Recipient Oocytes for Somatic Cell Cloning

Retrieval, selection for extracorporeal meiotic maturation, and* in vitro* culture of pig oocytes for the purposes of SCNT were accomplished according to the methods applied by Samiec and Skrzyszowska [16], Samiec et al. [22], and Skrzyszowska et al. [20]. Briefly, slaughterhouse ovaries were collected from both prepubertal female pigs (gilts) and postpubertal female pigs (gilts and sows). Cumulus-oocyte complexes (COCs) were recovered by aspiration of follicular fluid from 2 to 6 mm antral ovarian follicles. The COCs, with evenly granulated ooplasm and several uniform layers of compact cumulus cells, were selected for* in vitro* maturation. The maturation medium was comprised of HEPES- and NaHCO_3_-buffered Tissue Culture Medium 199 (TCM 199) that was supplemented with 10% porcine follicular fluid (pFF), 10% FBS, 5 ng/mL recombinant human basic fibroblast growth factor (rh-bFGF), 10 ng/mL recombinant human epidermal growth factor (rhEGF), 0.6 mM* L*-cysteine, 1 mM dibutyryl cyclic adenosine monophosphate (db-cAMP; bucladesine), 0.1 IU/mL human menopausal gonadotropin (hMG), and 5 mIU/mL porcine follicle-stimulating hormone (pFSH). Approximately 50 to 60 COCs were cultured in the db-cAMP- and hMG + pFSH-supplemented medium for 20 h at 39°C in a 100% water-saturated atmosphere of 5% CO_2_ and 95% air. The oocytes were then cultured for 22 to 24 h in fresh maturation medium that did not contain db-cAMP, hMG, or pFSH. After maturation, expanded cumulus cells and corona cells were completely removed by vigorous pipetting of the COCs in the presence of 0.1% hyaluronidase for 1 to 2 min. The metaphase II-stage oocytes, which had been selected on the basis of accepted morphological criteria involving evenly granulated, dark ooplasm and the presence of distinctly extruded first polar bodies, provided a source of host cytoplasm for the cell nuclei of TSA-exposed or TSA-unexposed bone marrow-derived MSCs in the somatic cell cloning procedure.

### 2.6. Production of Porcine Nuclear-Transferred Embryos Using MSCs Modulated or Not Modulated Epigenomically by TSA Treatment

The approaches to somatic cell cloning and* in vitro* culture of nuclear-transferred pig embryos that were used in the current experiments were thoroughly presented in our previous studies [15, 16, 18, 59, 60]. Concisely, prior to the SCNT procedure, cumulus-denuded* in vitro* matured gilt/sow oocytes were incubated in the maturation medium supplemented with 0.4 *μ*g/mL demecolcine (DMCC) for 50 to 60 min. The DMCC-treated oocytes were subsequently transferred into a micromanipulation chamber filled with TC 199 medium containing 4 mg/mL bovine serum albumin (fraction V; BSA-V) and 7.5 *μ*g/mL cytochalasin B (CB). Metaphase chromosomes, which had been allocated into the chemically induced protrusion of the plasma membrane, were removed microsurgically. The chemically assisted enucleation was accomplished by gently aspirating the ooplasmic cone, which contained the condensed chromosome cluster, with the aid of a bevelled micropipette. The reconstruction of enucleated oocytes was achieved by their electrofusion with TSA-treated or TSA-untreated MSCs. Single nuclear donor cells were inserted into the perivitelline space of previously enucleated oocytes (i.e., ooplasts/cytoplasts). The resulting somatic cell-ooplast couplets were placed into a fusion/activation chamber filled with electroporation medium (dielectric solution). SCNT-derived oocytes were artificially stimulated using the protocol of simultaneous fusion and electrical activation (SF-EA). In the SF-EA protocol, electric pulses that induced a fusion of MSC-cytoplast couplets were simultaneously the stimuli initiating the activation of reconstructed oocytes. The complexes of ooplasts and MSCs were subjected to plasma membrane electroporation by application of two successive DC pulses of 1.2 kV/cm for 60 *μ*s. The electropermeabilization of cell plasma membranes was performed in an isotonic dielectric solution (0.3 M* D*-mannitol) with concentration of CaCl_2_ increased up to 1.0 mM. Following SF-EA, porcine cloned embryos were exposed to 5 *μ*g/mL CB for 2 h and subsequently cultured* in vitro* in North Carolina State University-23 (NCSU-23) medium supplemented with 4 mg/mL BSA-V, 1% Eagle's Minimum Essential Medium-nonessential amino acid solution (MEM-NEAA), and 2% MEM-essential amino acid solution (MEM-EAA) for 72–96 h. Afterwards, cleaved embryos were cultured in NCSU-23/BSA-V + MEM-NEAA + MEM-EAA medium supplemented with 10% FBS for an additional 72 h up to morula and blastocyst stages.

### 2.7. Assessment of Cloned Blastocyst Quality by TUNEL Assay

The blastocysts developed from NT oocytes reconstructed with TSA-treated and -untreated MSCs were analyzed using a Deadend Fluorometric TUNEL System (Promega; Warsaw, Poland) according to the protocol of terminal deoxynucleotidyl transferase- (TdT-) mediated dUTP (2′-deoxyuridine-5′-triphosphate) nick-end labelling (TUNEL) that was dependent on the fluorescein isothiocyanate (FITC) conjugated with dUTP. This protocol was thoroughly described by Opiela et al. [61, 62]. Briefly, after fixation with 1% paraformaldehyde (PFA) diluted in PBS solution, the embryos were rinsed extensively (3 times). Subsequently, they were exposed to 0.2% Triton X-100 solution for 5 min, followed by 1 h incubation in the reaction mixture consisting of equilibration buffer, a cocktail of nucleotides, and TdT enzyme at a maximum humidity. Finally, the embryos were placed into a 2x concentrated solution of saline/sodium chloride-sodium/trisodium citrate (SSC) buffer for 15 min. After all the incubations carried out, cloned blastocysts were washed three times in PBS/polyvinylpyrrolidone (PVP) solution for at least 5 min and lastly transferred into a drop of VECTASHIELD mounting medium. This medium was supplemented with 4′,6-diamidino-2-phenylindole (DAPI) counterstain and has been found to be unsurpassed in preventing photobleaching of DAPI fluorochrome in order to retain antifading ability during long-term storage of dyed embryos. The evaluation of embryos that had been subjected to TUNEL analysis was performed under a fluorescence microscope (Nicon Eclipse E600, Tokyo, Japan). The assessment of blastocyst cytological quality enabled visualizing the total number of blastomere nuclei in each embryo and the number of cell nuclei exhibiting internucleosomally fragmented DNA, into which a FITC-conjugated dUTP was incorporated. The fluorescence filters adjusted to excitation wavelength higher than 460 nm and the filters adapted to emission wavelengths ranging from 480 nm to 540 nm were applied to detect the blue fluorescence emitted by cell nuclei of all the DAPI-tagged blastocyst blastomeres and the FITC-derived green fluorescence emitted by cell nuclei of late-apoptotic blastomeres.

### 2.8. Determination of Apoptotic Index (DCI) in Cloned Blastocysts

All the blastocysts generated were subjected to TUNEL analysis. The number of all DAPI-dyed blastomere nuclei (i.e., the total number of inner cell mass/ICM and trophoblast cell nuclei) and the number of all TUNEL-positive/FITC-stained (i.e., apoptotic) cell nuclei were estimated for each embryo. To assess the apoptotic index (dead cell index; DCI) in the single blastocyst, the sum of all apoptotic cell nuclei detected in the analyzed blastocyst was divided by the sum of all ICM/trophoblast cell nuclei identified in this blastocyst and subsequently multiplied by 100 [62].

### 2.9. Analysis of Porcine Cloned Embryos for* Oct4*,* Nanog*, and* Nestin* mRNA Expression Profile Using Quantitative Reverse Transcriptase Real-Time PCR (qRT-PCR)

The qRT-PCR was applied to estimate the expression levels of mRNA transcripts for pluripotency-related target genes (*Oct4*,* Nanog*) and multipotent stemness-associated target gene (*Nestin*) in two groups of SCNT-derived blastocysts encompassing the embryos developed from oocytes reconstructed with either TSA-treated or -untreated MSC nuclei. Briefly, 40 *μ*L of SideStep Lysis & Stabilization Buffer (Agilent Technologies) was added to 10 *μ*L of blastocysts sample (10 blastocysts) in PBS. The SideStep Buffer was used to lyse the cells of blastocysts and ensure RNA stabilization. The lysed embryo samples were vortexed at room temperature for 1 min. The one-step Brilliant II SYBR Green QRT-PCR Master Mix Kit (Agilent Technologies) was used to perform relative quantification of gene expression. Each PCR probe (total volume of 25 *μ*L) was comprised of 1 *μ*L of embryo cell lysate and 24 *μ*L of reaction mixture, which consisted of 12.5 *μ*L 2x SYBR Green QRT-PCR master mix (containing an optimized RT-PCR buffer, MgCl_2_, nucleotides (GAUC), SureStart* Taq* DNA polymerase, ^†^SYBR Green, and stabilizers) and 0.4 *μ*L of each 200 nM forward and reverse primer (Table 1), 1 *μ*L of RT/RNase block enzyme mixture, and 9.7 *μ*L RNase-free water. Thermal cycling conditions were as follows: 30 min at 50°C (for the first-strand synthesis); 10 min at 95°C; 40 cycles of 30 s at 95°C for denaturing, 60 s at 57°C for annealing, and 30 s at 72°C for extension.

The glyceraldehyde-3-phosphate dehydrogenase (*GAPDH*) housekeeping gene was used as an endogenous standard. The results for expression levels/profiles of individual target genes (*Oct4*,* Nanog*, and* Nestin*) were normalized according to the relative concentration of the endogenous standard. Each reaction was run in triplicate and the obtained results were averaged. By sample we mean mRNA derived from a pool of 10 blastocysts. We made six PCR runs from one mRNA sample: 3 for three analyzed genes (*Oct4, Nanog*, and* Nestin*) and 3 for the* GAPDH *control gene. Each cDNA synthesis and PCR amplification were achieved in one tube and one buffer. All data are presented as the mean values of the relative abundance (RA) of* Oct4, Nanog*, and* Nestin *mRNA transcripts. Expression of these transcripts isolated from blastocysts that originated from TSA-treated and -untreated groups was compared to that for control blastocysts, which served as a calibrator. Experiments were carried out on a Mastercycler Realplex (Eppendorf, UK Limited, Cambridge).

### 2.10. Statistical Analysis

The *χ* ^2^ test was applied to estimate the differences in the* in vitro* developmental capabilities of porcine NT embryos originating from TSA-treated and -untreated MSCs. The statistical analysis of variance (ANOVA) and Tukey's Honestly Significant Difference (HSD) post hoc test for multiple ranges were used for comparison of the levels of* Oct4, Nanog*, and* Nestin* transcripts between blastocysts derived from oocytes reconstituted with the cell nuclei of TSA-exposed and -unexposed MSCs. The ANOVA and subsequent Tukey's HSD post hoc test were also applied to compare the mean values calculated for total number of DAPI-labelled cell nuclei, total number of TUNEL-positive/FITC-labelled (i.e., apoptotic) cell nuclei, and apoptotic index (DCI) between blastocysts developed from NT oocytes generated using epigenetically modulated and nonmodulated MSCs. The differences with a probability (*P*) less than 0.05 were considered to be significant.

## 3. Results

### 3.1. The* In Vitro* Developmental Potential of Cloned Embryos Derived from the Cell Nuclei of MSCs Undergoing or Not Undergoing TSA-Based Epigenomic Modulation

**Figure 1:**
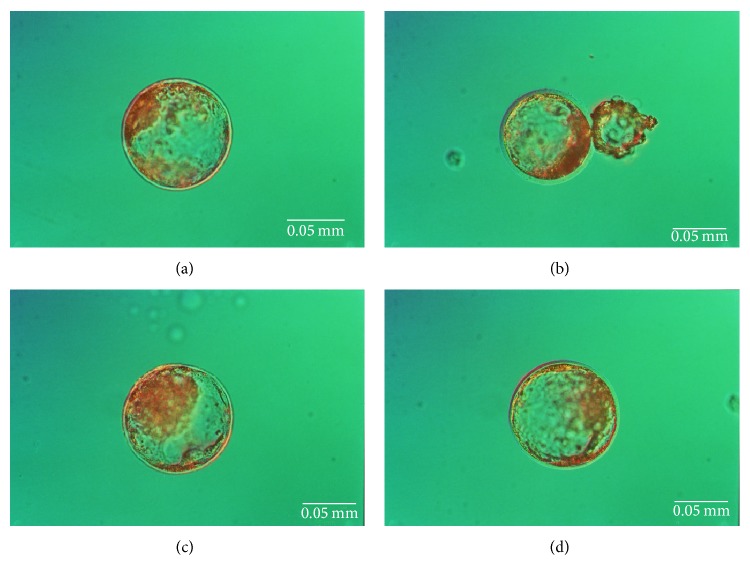
Porcine cloned blastocysts developed from nuclear-transferred oocytes reconstituted with adult bone marrow-derived mesenchymal stem cells undergoing trichostatin A- (TSA-) dependent epigenomic modulation (photographs (a) and (b)) or not undergoing TSA-dependent epigenomic modulation (photographs (c) and (d)). Images were taken at magnification ×200.

Although the cleavage activity did not vary between cultured nuclear-transferred embryos reconstituted from MSCs subjected and not subjected to TSA-dependent epigenetic transformation (*P* ≥ 0.05), the morula and blastocyst formation rates were characterized by statistically significant differences. A significant variability in the* in vitro* developmental competences to reach the morula stage (*P* < 0.05) and a very highly significant variability in the developmental competences to reach the blastocyst stage (*P* < 0.001; Figure 1) were observed between these two groups of cloned embryos, respectively. The detailed data that are focused on the assessment of extracorporeal developmental capability of NT embryos reconstituted with epigenetically nonmodulated or modulated MSCs are presented in Table 2. As compared to the present investigation, in our previous studies, in which the* in vitro* developmental potential of cloned pig embryos derived from the cell nuclei of fetal fibroblast cells (being currently used source of nuclear donor cells) and generated applying the same experimental protocols of SF-EA for artificial stimulation of nuclear-transferred oocytes was explored, the percentages of the obtained morulae and blastocysts ranged from 58.2% to 61.3% and from 29.6% to 33.2%, respectively [16, 18, 20]. It is worth noting that all these percentages turned out to be considerably lower than the morula and blastocyst formation rates achieved for NT embryos produced using epigenomically nontransformed or transformed MSCs (Table 2).

### 3.2. Dependence of the Cloned Blastocyst Quality Evaluated by TUNEL Assay on the TSA-Based Epigenomic Modulation of MSCs

**Figure 2:**
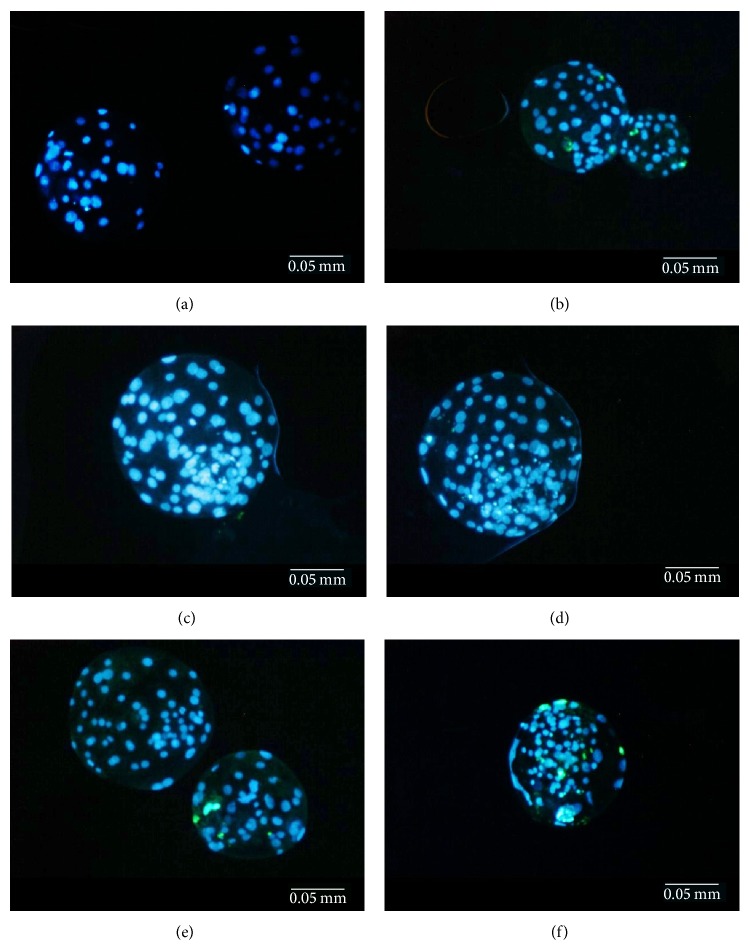
Evaluation of cytological quality of porcine cloned blastocysts on the basis of simultaneous determination of total nuclear number and detection of apoptotic cell nuclei by terminal deoxynucleotidyl transferase- (TdT-) mediated dUTP (2′-deoxyuridine-5′-triphosphate) nick-end labelling (TUNEL) analysis. Photographs (a) to (d) depict embryos originating from nuclear-transferred (NT) oocytes reconstituted with adult bone marrow-derived mesenchymal stem cells (ABM-MSCs) subjected to epigenetic transformation* via* trichostatin A (TSA) treatment. Photographs (e) and (f) depict embryos originating from NT oocytes reconstituted with ABM-MSCs not subjected to epigenetic transformation* via* TSA treatment. In each blastocyst, the cell nuclei of all the blastomeres (both inner cell mass (ICM) and trophectoderm (TE) cells) had been tagged with 4′,6-diamidino-2-phenylindole (DAPI) counterstain and subsequently fluoresced in blue. The cell nuclei of late-apoptotic blastomeres (ICM and/or TE cells) exhibiting internucleosomal DNA fragmentation had been dyed with fluorescein isothiocyanate (FITC) and then fluoresced in bright green. In each photograph, the DAPI-derived blue and FITC-derived green fluorescent signals merge into one another. Photographs (a) to (f) represent the blastocysts displaying different incidence of blastomere apoptosis and thereby varied advancement of internucleosomal DNA fragmentation ((a), (c), (d), and (e) the lack of apoptotic intranuclear DNA fragmentation; (b) and (e) few apoptotic cell nuclei; (f) increased extent of apoptotic intranuclear DNA fragmentation). Images were taken at magnification ×200.

The quality of nuclear-transferred embryos derived from TSA-exposed MSCs was considerably higher than that of NT embryos derived from TSA-unexposed MSCs. Very highly significant differences in the mean number of DAPI-stained cell nuclei per blastocyst were shown between these two groups of embryos (*P* < 0.005; Table 3, Figure 2). In turn, no significant differences in the mean number of TUNEL-positive (i.e., late-apoptotic) cell nuclei per blastocyst were identified between NT embryos reconstituted with MSCs undergoing and not undergoing TSA-mediated epigenetic transformation (*P* ≥ 0.05; Table 3, Figure 2). Although no significant differences in the DCI per blastocyst were found between both experimental groups (*P* ≥ 0.05), the incidence of late apoptosis-related internucleosomal DNA fragmentation trended slightly upwards for NT embryos generated using TSA-untreated MSCs as compared to those produced using TSA-treated MSCs (Table 3, Figure 2).

### 3.3. The Pluripotency Status of Porcine Nuclear-Transferred Embryos Originating from Epigenomically Modulated or Nonmodulated MSCs

The total number of analyzed cloned blastocysts was 60. To assess relative abundance (RA) of three analyzed mRNA transcripts for pluripotency-related target genes (*Oct4*,* Nanog*), multipotent stemness-associated target gene (*Nestin*), and control* GAPDH* gene transcript, we used 3 × 10 blastocysts from both TSA-treated and -untreated groups.

**Figure 3:**
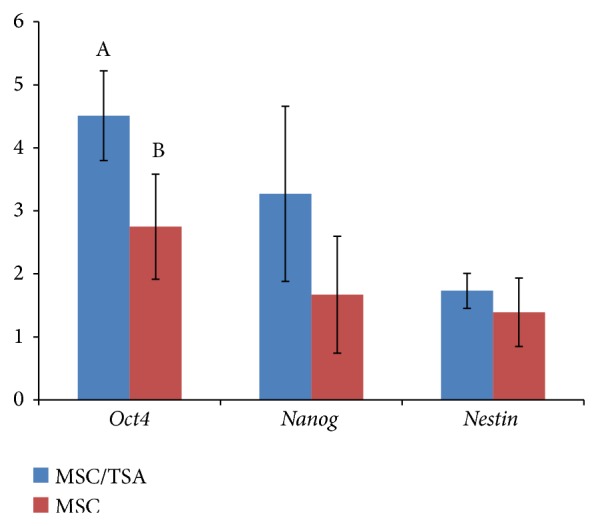
Relative abundance (mean ± SD) of* Oct4*,* Nanog*, and* Nestin *mRNAs in blastocysts developed from nuclear-transferred pig embryos descended from MSCs undergoing or not undergoing TSA-dependent epigenomic transformation. The highly significant differences were designated with the A and B letters (*P* < 0.01; ANOVA followed by Tukey's HSD post hoc test).

Highly significant differences were indicated for the* Oct4* gene transcript quantitative profile between blastocysts originating from TSA-treated and -untreated groups (*P* < 0.01; Figure 3). Although no significant intergroup variability was shown in the RA of* Nanog *and* Nestin* mRNAs (*P* ≥ 0.05), their expression levels tended to be higher in blastocysts derived from NT embryos reconstructed with TSA-exposed MSCs as compared to the TSA-untreated group (Figure 3).

## 4. Discussion

The abundance of the morulae and blastocysts of higher cytological and molecular quality, generated in the present investigation, confirms that the reprogrammability of adult bone marrow-derived mesenchymal stem cells, which had been epigenetically modified* via* exposure to trichostatin A, underwent considerable improvement in a cytoplasm of porcine nuclear-transferred oocytes and resultant* in vitro* cultured cloned embryos. It is beyond any doubt that the impact of TSA-based epigenomic transformation of bone marrow-retrieved MSCs, representing multipotent and undifferentiated stem cells, on their competence for SCNT has not yet been explored not only in pigs, but also in other mammalian species. So far, TSA-mediated epigenetic modulation has been applied only for differentiated somatic cells that commonly provide the source of nuclear donors for cloning procedure in pigs. The results of the study by Diao et al. [55] proved that porcine NT embryos reconstituted with TSA-treated fetal fibroblast cells were characterized by twofold higher developmental potential to reach the blastocyst stage (30%) than the NT embryos reconstituted with TSA-untreated fibroblast cells (15%). Similar tendency has been also shown in our current study, in which trichostatin A-mediated epigenetic transformation of bone marrow-derived MSCs gave rise to significant enhancement of capability of cloned embryos to complete their development to blastocyst stage (65%) as compared to that observed for embryos derived from TSA-unexposed MSC nuclei (46%). Nonetheless, NT pig embryos originating from epigenomically transformed fetal fibroblast cells displayed considerably lower blastocyst formation rate [55] than the rate indicated in our present study for NT embryos reconstructed with epigenomically nontransformed MSCs. Moreover, treatment of nuclear donor MSCs with trichostatin A resulted in one and a half times to severalfold increase of blastocyst percentage (65%) as compared to the blastocyst yields obtained by other investigators using undifferentiated or* in vitro* differentiated MSCs that were not modulated epigenetically [26–28, 31, 32]. In the study by Kumar et al. [28], the abilities of epigenomically nontransformed bone marrow-descended MSC nuclei to direct the* in vitro* development of porcine cloned embryos to blastocyst stage decreased above three times (approximately 20%) as compared to our present work. In turn, nuclear-transferred embryos that had originated from porcine undifferentiated bone marrow-retrieved MSCs and their derivatives along the osteogenic lineage were able to complete their* in vitro *development to blastocyst stage at the rates ranging from 33% to 45% [31]. In contrast, Jin et al. [32] and Li et al. [27] reported that blastocyst yields of NT embryos derived from porcine epigenetically unmodulated MSCs were maintained at the relatively low levels of approximately 18% and 16%, respectively. Nevertheless, in the study by Lee et al. [26], the* in vitro* developmental outcome to the blastocyst stage of NT embryos originating from undifferentiated MSCs achieved the rate of approximately 48%, but even this blastocyst formation rate was considerably lower than that noticed in our current investigation.

The results of the current study have also clearly demonstrated that the TSA-dependent epigenomic transformation of nuclear donor MSCs contributes to improvement of not only* in vitro* developmental competences, but also quality and transcription level-related pluripotency extent of porcine cloned embryos. The utilization of undifferentiated mesenchymal stem cells and the modification of the molecular mechanisms of transcriptional reprogramming of the donor nuclear genome* via* HDAC inhibitor-mediated epigenetic modulation of MSCs before their use for SCNT led to correct and complete adaptation of MSC-inherited genomic DNA to the cytoplasmic environment of enucleated oocytes and* in vitro* cultured NT pig embryos. Epigenetic reprogramming of donor cell nuclei suggests that a new program for their transcriptional activity is loaded and reloaded immediately following reconstruction of enucleated oocytes. The success of SCNT may depend upon both genomic DNA-associated reprogramming of gene expression for dedifferentiation of the donor somatic cell nuclei during early preimplantation development of cloned embryos and reprogramming of gene expression for onset of somatogenic nuclear redifferentiation during blastocyst formation [7, 10, 63, 64]. It has been ascertained that somatic cell nuclei should undergo the wide DNA cytosine residue demethylation changes throughout the early development of NT embryos to erase and then reset their own overall epigenetic as well as parental genomic imprinting memory, which has been established by remethylation of the nuclear genome within the framework of the specific pathway of somatic and germ cell lineage commitment and differentiation [2, 4, 13, 30, 65–67].

It appears that the enhanced cytological quality of cloned pig blastocysts that originated from enucleated oocytes receiving epigenetically modulated MSC nuclei could be associated with more faithful and faultless pattern for reprogramming of transcriptional activity of genomic DNA inherited from undifferentiated and multipotent stem cells. These cells exhibit increased genomic and epigenomic plasticity in rearrangements of their gene expression in the blastomeres of preimplanted NT embryos. The fluorocytochemical analysis of overall mean nuclear counts revealed almost twofold higher total cells number in porcine blastocysts developed from NT oocytes reconstituted with TSA-exposed MSCs (48 blastomeres) than that identified in blastocysts derived from NT oocytes reconstituted with TSA-unexposed MSCs (28 blastomeres). The quality of cloned blastocysts generated in our present study using epigenomically transformed MSCs was comparable to that noticed in the study by Lee et al. [26] for porcine NT blastocysts derived from epigenomically nontransformed MSCs. These blastocysts were also characterized by a total cells number equal to 48. In contrast to the abovementioned findings, in the investigations carried out by Li et al. [27], Kumar et al. [28], and Jin et al. [32], the overall number of ICM and trophectoderm cells in cloned pig blastocysts originating from embryos reconstituted with TSA-untreated MSCs ranged from 28 to 35 and decreased approximately one and a half times as compared to the total cells number estimated for NT blastocysts obtained by us in the TSA-exposed MSC group. Nevertheless, the mean apoptotic index (i.e., DCI = 4.95%) that was calculated by us for blastocysts developed from cloned embryos reconstituted with epigenetically modified MSC nuclei was similar to the ratios of TUNEL-positive cells (4.6 to 4.7%) observed by Jin et al. [32] and Kumar et al. [28] among porcine NT blastocysts derived from epigenetically nonmodulated counterparts. In turn, cloned blastocysts produced in our study using TSA-untreated MSCs displayed the apoptotic index (7.13%) that was comparable or slightly lower to indexes identified for cloned embryos created with the aid of fetal fibroblast cells not undergoing exposure to trichostatin A [28, 32]. The latter indexes (DCIs) oscillated between 7.3 and 7.8%. Generally, in our current work, the proportion of TUNEL-positive cells in relation to a total cells number tended to insignificantly decrease among NT blastocysts generated using undifferentiated mesenchymal stem cells subjected to TSA treatment compared to those generated using nuclear donor cells not exposed to TSA (4.95%* versus* 7.13%). Analogous slight downward tendency in the ratios of TUNEL-positive cells was indicated by Diao et al. [55], comparing blastocysts developed from cloned embryos reconstructed with cell nuclei of fetal fibroblasts treated with TSA and the counterparts produced using TSA-unexposed fetal fibroblast cell nuclei (nearly 3%* versus* 4.5%).

Improved reprogrammability of transcriptional activity for nuclear genome of epigenetically modulated MSCs in the cells of preimplanted NT embryos turned out to be positively correlated with enhanced molecular quality of porcine cloned blastocysts assessed on the basis of their pluripotency extent, which was measured with the expression profiles identified for* Oct4* and* Nanog* genes. A 38-kDa protein Oct4 (i.e., octamer-binding transcription factor 4) that is a member of the family of POU- (Pit-Oct-Unc-) domain and homeodomain transcription factors acts as a vital regulator of pluripotency extent, playing an important role in not only controlling preimplantation embryonic development, but also maintenance of ICM cell fate in blastocysts and pluripotency status of embryonic stem cells (ESCs) [68–70]. A 35-kDa protein designated as Nanog from Celtic/Irish mythical* Tír na nÓg* (Tir Na Nog; The Land of the Ever-Young) is another homeobox-containing transcription factor that represents the group of pivotal proteins modulating pluripotency degree [70, 71]. The homeoprotein Nanog can act synergistically with Oct4 protein in retaining the pluripotent status of blastocyst-descended ICM and epiblast cells as well as in sustaining the undifferentiated status and ability for self-renewal of ESCs [71, 72]. The aberrant (i.e., downregulated) expression levels and patterns of* Oct4* and* Nanog* transcripts in both bovine NT blastocysts produced using epigenetically nonmodulated calf dermal fibroblast cells [73] and porcine NT blastocysts produced using either epigenetically nonmodulated adult MSCs or fetal fibroblast cells [28] have been found to be a major cause of not only their declined cytological and molecular quality, but also decreased* in vitro *developmental potential of cloned embryos. However, in our current study, it seems that TSA-dependent epigenomic transformation of nuclear donor MSCs biases correctingly the expression profile of* Oct4 *and* Nanog* mRNAs in cloned pig blastocysts, triggering both significant enhancement of relative abundance (RA) of* Oct4* transcripts and slight (nonsignificant) increase in* Nanog* transcript RA as compared to the TSA-untreated MSC group. On the one hand, this influence could be exerted by direct diminishment in the deacetylation level within the nucleosomal core-derived histone lysine moieties that was evoked by trichostatin A-mediated nonspecific inhibition of HDACs. On the other hand, it could be probably elicited* via* indirect nonselective suppression of cytosine residue methylation processes within the* Oct4* and* Nanog* gene promoters and/or enhancers, leading to upregulated expression of these crucial pluripotency-related genes. Similar results have been shown in the study by Wang et al. [73], in which the sequential treatment of bovine differentiated nuclear donor fibroblast cells and resultant cloned embryos by both nonspecific inhibitors of DNMTs and HDACs (i.e., 5-aza-dC and TSA, resp.) contributed to increase of* Oct4* transcript RA in the blastocysts obtained, simultaneously sustaining the expression of* Nanog *mRNAs on the unchanged level as compared to the 5-aza-dC- and TSA-unexposed group. In this case, 5-aza-2′-deoxycytidine- and trichostatin A-mediated epigenetic modulation of both nuclear donor cells and cloned embryos gave rise presumably to direct downregulation in the methylation of cytosine residues within the* Oct4* gene promoter and/or enhancer that resulted from 5-aza-dC-dependent nonspecific inhibition of DNMTs. Furthermore, this two-factor epigenetic modulation could affect the enhanced incidence of acetylation level within the nucleosomal core-derived histone lysine moieties that was triggered by trichostatin A-mediated nonselective inhibition of HDACs, consequently leading to indirect upregulated expression of the* Oct4* gene. Taking into consideration all the abovementioned findings, considerable transcriptional upregulation in the expression of* Oct4* gene (confirmed among porcine NT blastocysts in our present study and among bovine NT blastocysts in the study by Wang et al. [73]) can be found to be pivotal indicator of increased pluripotency extent of cloned embryos. This process appears to be also associated with improved cytological quality of NT blastocysts that was measured with their total cell counts. In the current investigation, we have shown that the overall number of ICM and trophoblast cells in blastocysts was significantly higher following the reconstruction of NT pig embryos with epigenomically transformed mesenchymal stem cells.

## 5. Conclusions and Future Goals

Summing up, artificial epigenomic modulation of* in vitro* cultured MSCs using the nonspecific HDAC inhibitor, designated as TSA, seems to facilitate much more the reprogramming process for epigenetically determined transcriptional activity of somatic cell-inherited nuclear genome in the NT pig embryos. The use of ectopic HDAC inhibitors for epigenetic transformation of mesenchymal stem cells, whose cell nuclei were transferred into enucleated oocytes, is the completely new approach in the studies involving somatic cell cloning of pigs and other mammalian species. Enhanced reprogrammability of nuclear genome descended from TSA-exposed MSCs in the blastomeres of porcine preimplanted cloned embryos resulted in the improvements of not only their capacity to complete* in vitro* development to the morula and blastocyst stages, but also cytological and molecular quality of the blastocysts produced. Therefore, further investigations are also necessary to determine whether the novel strategy of TSA-dependent epigenetic modification of nuclear donor MSCs, which has been recently utilized in our laboratory for the future goals of generating cloned piglets, enables retaining the* in vivo *developmental competences of the high-quality NT-derived blastocysts to reach full term.

## Figures and Tables

**Table 1:** Primers used in quantitative reverse transcriptase real-time PCR (qRT-PCR).

Gene	Forward/reverse	Primer	Size (bp)
*Nanog *	Forward	5′-GCTCTGTGTCCTCAACGACA-3′	169
	Reverse	5′-GCTATTCCTTGGCCAGTGGT-3′	

*Nestin *	Forward	5′-TGAAGCCAAGGTGGTCATCC-3′	150
	Reverse	5′-TTGACCTCTAAGCTGTGGCG-3′	

*Oct4 *	Forward	5′-AGTGAGAGGCAACCTGGAGA-3′	152
	Reverse	5′-CACTGCTTGATCGTTTGCCC-3′	

*GAPDH *	Forward	5′-GGGCATGAACCATGAGAAGT-3′	133
	Reverse	5′-TGTGGTCATGAGTCCTTCCA-3′	

**Table 2:** Effect of the TSA-dependent epigenomic modulation of MSCs on the *in vitro* developmental outcome of cloned pig embryos.

TSA-mediated epigenetic transformation of MSCs	Number of oocytes/embryos			Development to	
	Enucleated	Electrofused (%)	Cleaved (%)	Morulae (%)	Blastocysts (%)
+	186	178/186 (95.7)	174/178 (97.8)	155/178 (87.1)^a^	116/178 (65.2)^A^
−	293	275/293 (93.9)	262/275 (95.3)	216/275 (78.5)^b^	125/275 (45.5)^B^

TSA: trichostatin A; MSC: mesenchymal stem cell.

Values with different small superscript letters (a and b) within the same column denote statistically significant differences between experimental groups (*P* < 0.05; *χ* ^2^ test). Values with different large superscript letters (A and B) within the same column denote very highly significant differences (*P* < 0.001; *χ* ^2^ test). Number of replicates ≥6.

**Table 3:** Effect of the TSA-mediated epigenomic modulation of MSCs on the cytological quality of cloned pig embryos assessed by TUNEL assay.

TSA-mediated epigenetic transformation of MSCs	Number of analyzed blastocysts	Mean number of DAPI-tagged cell nuclei per blastocyst ± SD	Mean number of TUNEL-positive (apoptotic) cell nuclei per blastocyst ± SD	Mean apoptotic index (DCI) per blastocyst ± SD (%)
**+**	31	47.97 ± 27.43^A^	1.61 ± 3.38	4.95 ± 13.98
−	26	27.77 ± 15.82^B^	1.58 ± 2.69	7.13 ± 12.44

TSA: trichostatin A; MSC: mesenchymal stem cell; TUNEL: terminal deoxynucleotidyl transferase- (TdT-) mediated dUTP (2′-deoxyuridine-5′-triphosphate) nick-end labelling; DAPI: 4′,6-diamidino-2-phenylindole; SD: standard deviation; DCI: dead cell index.

Values with different large superscript letters (A and B) within the same column denote very highly significant differences (*P* < 0.005; ANOVA followed by Tukey's HSD post hoc test). Number of replicates = 6.
